# Radiotherapy Compared to Other Strategies in the Treatment of Stage I/II Follicular Lymphoma: A Study of 404 Patients with a Median Follow-Up of 15 Years

**DOI:** 10.1371/journal.pone.0131158

**Published:** 2015-07-06

**Authors:** Dlawer Abdulla Barzenje, Milada Cvancarova Småstuen, Knut Liestøl, Alexander Fosså, Jan Delabie, Arne Kolstad, Harald Holte

**Affiliations:** 1 Department of Oncology, Ostfold Hospital Trust, Fredrikstad, Norway; 2 Department of Biostatistics, Oslo University Hospital, Oslo, Norway; 3 Center for Cancer Biomedicine, University of Oslo, Oslo, Norway; 4 Department of informatics, University of Oslo, Oslo, Norway; 5 Department of Oncology, Oslo University Hospital, Oslo, Norway; 6 Department of Pathology, Oslo University Hospital, Oslo, Norway; National Cancer Center, JAPAN

## Abstract

**Purpose:**

To investigate outcome for patients with follicular lymphoma (FL) stage I-II treated at a population-based referral institution with a median follow-up of 15 years. Overall and cause-specific survival was compared to that of a sex, age and residency matched individuals from normal population.

**Material and Methods:**

404 patients with early stage FL treated between 1980 and 2005 were retrospectively analyzed. Two of three patients had stage I disease. Based on clinical characteristics, first line treatments were radiotherapy (RT) (48% of patients), chemotherapy (CT) (16%), combined chemo-and radiotherapy (CRT) (16%) or observation (OBS) (15%). Survival was modeled with Kaplan-Meier methodology. Multivariate analyses were performed with the Cox model.

**Results:**

Fifteen years overall survival (OS), progression free survival (PFS) and time to next treatment (TNT) were 50% (95% confidence interval [CI]: 45–55), 42% (95% CI: 36–47) and 48% (95% CI, 42–54), respectively. For patients treated with RT 97% achieved a complete remission, and 15 year OS, PFS and TNT were 57% (95% CI, 50–64), 46% (95% CI, 39–54) and 49% (95% CI, 42–57), respectively. Relapse rate after RT and CRT was 49% and 36%, respectively. Only 2% of patients who received RT or CRT relapsed inside the radiation field and 5% had isolated near-field relapse. No statistical differences were found between treatment groups regarding death from cardiovascular disease or incidence of second cancer. Compared to a matched normal population, non-lymphoma cancer mortality was higher among patients given RT, hazard ratio 1.66 (95% CI: 1.14–2.42; P<0.01). Compared to other treatment modalities, patients selected for observation without treatment did not have inferior outcome.

**Conclusions:**

A differentiated treatment strategy in early stage FL results in long term survival for the majority of patients. OBS is a valid initial choice for selected patients without lymphoma-related symptoms.

## Introduction

Malignant lymphomas constitute approximately 4% of all cancers in the Western world annually, and the incidence is increasing. Follicular lymphoma (FL) is the second most common type of non-Hodgkin lymphoma (NHL) representing 20–25% of cases. Almost one third of patients with FL present in stage I or II at the time of diagnosis [1].

In accordance with international recommendations, patients with indolent lymphomas stage I or II with involvement of two (and in some cases three) adjoining lymph node regions have been treated with local radiotherapy (RT) at the Norwegian Radium Hospital (NRH) for the last three decades. Chemotherapy (CT) and combination of chemotherapy and radiotherapy (CRT) has been the preferred treatment for more widespread symptomatic stage II disease. The majority of patients in stage I for whom involved nodes have been removed surgically or patients with widespread non-symptomatic stage II disease have been observed (OBS) without initial treatment.

Previous studies have investigated outcome for patients with early stage FL and other indolent NHL including cause of death and incidence of second cancer. Reported 10 year rates of overall survival (OS) for patients with early stage FL treated with RT vary between 52–74% [2–8]. Combining chemotherapy and radiotherapy does not appear to improve OS [7, 9]. Hence, RT alone has by many been considered the standard approach for these patients. Lymphoma remains the leading cause of death in these studies. A study of secondary cancers showed no elevated cumulative incidence for patients receiving RT [4].

Our institution has a regional treatment responsibility for almost half the Norwegian population and serves as the only referral RT center for patients with malignant lymphoma within the region. A detailed clinical registry was established for all lymphoma patients in 1980. The population-based patient recruitment, the standardized treatment for early stage FL over several decades, the long follow-up, and the local high quality registry combined with available data from the Norwegian Cancer Registry and Statistics, Norway, made it possible to conduct a unique study on long term outcome for localized FL. Survival, cause of death and incidence of non-lymphoma cancer were compared to that of an age, sex and residency matched control population.

## Material and Methods

### Patients

Patients diagnosed with FL in stage I and II treated for the first time between January 1, 1980 and December 31, 2005 were selected. Staging procedures included patient history (B-symptoms or not), clinical examination and computed tomography or x-ray (prior to 1986, 28% of patients) of thorax, computed tomography of abdomen and pelvis, bone marrow biopsy, routine blood counts and biochemistry. Treatment and follow-up information were registered until death or December 31, 2012 for OS and December 31, 2011 for PFS and TNT.

### Ethics Statement

The study was approved by the Regional Committee for Medical and Health Research Ethics, South East, Norway. Written informed consent from participants was not mandatory. All patient records/information was anonymized and de-identified prior to analysis. We did not have any access to any personal information about matched normal population.

### Type of initial treatment

Modalities chosen for initial treatment and patient characteristics are shown in Table 1. Guidelines for treatment of early stage FL at our institution were:

1. Patients with stage I or stage II disease with two or three adjacent lymph node regions involved received RT.
2. Patients with stage II disease with involvement of non-adjacent lymph node regions received either CT or CRT or selected for OBS when asymptomatic.
3. Stage I patients after surgical removal of involved node or affected organ were also selected for OBS.

**Table 1:** Patient Characteristics.

		No.	%
**Total number**		404	100
**Gender**	Female	185	46
	Male	219	54
**Age in years**	Median (Range)	59 (18–87)	
**Stage** ^a^	I	210	52
	II/IIE	131	32
	I/extranodal	44	11
	II/extranodal	19	5
**Bulky**	Not bulky	352	87
	Bulky(max diameter ≥ 6cm)^b^	52	13
**FLIPI** ^c^	0–1	359	89
	2–3	33	8
**Extranodal involvement**	Absent	338	84
	Present	66	16
**B symptoms**	Absent	385	95
	Present	19	5
**WHO**	0	257	64
	1	143	35
	2–3	4	1
**Involved site**	Supradiaphragmatic	196	48
	Subdiaphragmatic	208	52
**Treatment**	RT	214	53
	CT	63	16
	CRT	64	16
	OBS	63	15
**Involved organ in I/extranodal**	Skin	12	
	Stomach/Intestine	12	
	Salivary gland	9	
	Thyroid gland	4	
	Oral/Nasal	2	
	Muscle/Tendon	2	
	Prostate/Testicle	2	
	Eye	1	
	Total	44	
**Involved organ in II/extranodal**	Skin	6	
	Oral/Nasal	5	
	Parotid gland	3	
	Stomach	2	
	Bone	2	
	Kidney	1	
	Total	19	

Comments:

^a^ Stage: II/IIE: Stage II with or without extranodal extension; I/extranodal: Stage I with primary extranodal involvement; II/extranodal: Stage II with primary extranodal involvement.

^b^ Maximum tumor diameter.

^c^ FLIPI was missing for 12 (3%) of patients.

Abbreviations: RT: Radiotherapy, CT: Chemotherapy, CRT: Chemotherapy followed by Radiotherapy, OBS: Observation without treatment.

RT was delivered as involved field photon beam irradiation including involved sites and the near-by draining lymph node region. Before 1998, the standard dose was 40 Gy / 20 fractions, thereafter 30 Gy / 15 fractions. Chemotherapy (CT) regimens were Chlorambucil/Prednisolone, CHOP (Cyclophosphamide, Doxorubicin, Vincristine, Prednisolone) and CVP (CHOP without Doxorubicin). Rituximab (R) was introduced early last decade and has been combined with chemotherapy as R-CHOP-21 (No = 11) or used as single agent (No = 4).

The term observation (OBS) is applied for patients with no immediate anti-lymphoma treatment within the first 3 months after diagnosis. For the 63 patients in the OBS group, 36 hade a stage I and underwent complete surgical removal. Type and number of interventions were excisional biopsy 26, gastrectomy 4, enterotomy 2, tonsillectomy 1, parotidectomy 1, thyroidectomy 1 and orchiectomy 1. The remaining 27 patients in the OBS group were all in stage II.

### Pathology and staging

The updated Kiel classification (1990–1994) [10], the R.E.A.L classification (1994–2001) [11] and the W.H.O classification (2001–2005) [12] were used for histological diagnosis. FL in the two former classifications, respectively called centroblastic/centrocytic or follicle center cell lymphoma was translated into the WHO classification. However, all cases with primary extranodal involvement were reviewed because of a potential confusion with marginal zone lymphoma. Also, all cases recorded with an uncertain diagnosis of follicular lymphoma were reviewed. In most cases, only histology using W.H.O criteria were used to review the cases and, where necessary, supplemented by a limited panel of antibodies including CD20, BCL6, BCL2 and CD10. As for cases reported with an uncertain diagnosis, the same was done. The total number of reviewed cases was 69 (17%). In addition 6 more patients were excluded from the study as they had other diagnosis (4 patients with marginal zone lymphoma, one with diffuse large B-cell lymphoma and one with follicular hyperplasia). Patients with a reported FL grade 3B were excluded.

The Rye staging system from 1966 with later modifications [13–15] was used throughout the study period. Stage I/extranodal and stage II/extranodal were defined as primary extranodal tissue involvement without or with regional lymph node involvement, respectively [14]. When analyzing data, stage I and stage I/extranodal, and stages II/IIE and II/extranodal were pooled as stage I (254 patients) and stage II (150 patients), respectively.

### Data collection

Data was retrieved from the Lymphoma registry, supplemented with data from the NRH RT registry, local hospitals and/or family doctors. Information about date and cause of death was updated from Statistics Norway. In addition, information on secondary cancer was retrieved from Norwegian Cancer Registry. Second cancer was defined as any malignant disease other than NHL which was diagnosed after primary lymphoma diagnosis.

The FL International Prognostic Index (FLIPI) was retrospectively determined for all patients [16] and performance status determined by WHO/ECOG (World Health Organization/Eastern Cooperative Oncology Group) criteria [17].

### Clinical endpoints and Statistical analyses

OS was calculated from date of initial diagnosis to death of any cause or end of follow-up on mortality, 31 Dec 2012, whichever came first. PFS was defined as time from date of diagnosis until date of relapse, death from lymphoma or end of follow-up on treatment and relapse 31 Dec 2011. TNT was defined as time from date of diagnosis until date of new therapy for first progression or relapse, or to the end of follow-up, whichever came first. [18]

Evaluation of response was done according to the Cheson criteria [18] and was carried out 1–3 months after end of primary treatment. All patients, including the OBS group, were then followed with regular visits every 2–6 months for a minimum of 5 years by our clinic, their local hospital or their general practitioner. Relapse was defined as a recurrence of lymphoma after initial complete remission (CR) or progression after either initial partial remission (PR) or stable disease (SD).

To compare OS of FL patients with the general population, each patient was matched with 5 individuals randomly selected from the general population. Controls were matched for age, sex and residency and had to be alive and cancer-free at the time of their case’s diagnosis. The match was performed by Statistics Norway.

Crude associations between pairs of categorical variables were assessed with Chi-square tests. Unadjusted survival curves were depicted using the Kaplan-Meier method and differences assessed with the log-rank test. Multivariate analyses were performed with Cox regression model and the results are expressed as hazard ratios (HR) with 95% confidence intervals (CI).

The Kaplan-Meier method was also used to compare OS for patients with controls. Risk of death due to other cancers and cardiovascular disease (CVD) were compared with the general population, adjusted to sex and modeled with the Cox regression method.

All tests were two-sided. P-values <0.05 were considered statistically significant. Statistical analyses were performed using IBM SPSS Statistics Version 22 and Stata version 13.

## Results

### Patient characteristics

A total of 404 patients with early stage FL were eligible for the study. Patient characteristics are listed in Table 1. Male to female ratio was 1.2. The majority of patients had a FLIPI score of 0–1, WHO performance status 0–1, no B-symptoms, non-bulky disease and primary nodal involvement. Median observation time for OS was 15 years (range 0.2–32.2).

Patient characteristics according to choice of initial treatment are described in Table 2. Type of initial treatment (RT, CT, CRT, OBS) was significantly associated with age </≥61 years (P = 0.002), WHO performance status 0 versus ≥1 (P<0.001), supradiaphragmatic versus subdiaphragmatic (P = 0.002), and stage I versus II (P<0.001). Thus, the majority of patients treated with RT had WHO status 0 and stage I disease while patients treated with CT or CRT more often presented with WHO status 1 or more, stage II and subdiaphragmatic disease. Most patients who received CRT belonged to the younger age group.

**Table 2:** Patient characteristics according to the type of initial treatment option.

			RT	CT	CRT	OBS	Total	*P* value (Chi-square)
**Gender**	Female	No.	98	31	23	33	185	0.272
		%	46	49	36	52		
	Male	No.	116	32	41	30	219	
		%	54	51	64	48		
**Age (years)**	18–60	No.	121	30	48	28	227	0.002
		%	56	48	75	44		
	≥ 61	No.	93	33	16	35	177	
		%	44	52	25	56		
**WHO**	0	No.	158	30	35	34	257	<0.001
		%	74	48	55	54		
	≥ 1	No.	56	33	29	29	147	
		%	26	52	45	46		
**FLIPI** ^a^	0	No.	100	22	28	21	171	0.245
		%	47	35	44	33		
	1	No.	96	31	28	33	188	
		%	45	49	44	53		
	≥ 2	No.	12	8	6	7	33	
		%	5	13	9	11		
**Bulky (Maximum diameter)**	< 6 cm	No.	198	46	46	62	352	<0.001
		%	92	73	72	98		
	≥ 6 cm	No.	16	17	18	1	52	
		%	8	27	28	2		
**Disease involvement**	Supradiaphragmatic	No.	120	20	24	32	196	0.002
		%	56	32	38	51		
	Subdiaphragmatic	No.	94	43	40	31	208	
		%	44	68	62	49		
**Stage**	I	No.	181	12	25	36	254	<0.001
		%	85	19	39	57		
	II	No.	33	51	39	27	150	
		%	15	81	61	43		

Comments:

^a^ FLIPI was missing for 12 (3%) of patients. Within each treatment group percentages are calculated vertically (column directions). Total numbers are calculated horizontally (row directions).

### Response to initial treatment

Response rates for the different types of initial treatment according to stage are shown in Table 3. The majority of patients (97%) who received RT as the single modality achieved complete remission (CR) regardless of stage. CR rates for patients who received CRT and CT alone were 81% and 65%, respectively.

**Table 3:** Response to different type of treatment and according to stage.

Treatment	Stage	Response			
		CR	PR	SD/PD	Total
		No. (%)	No. (%)	No. (%)	No. (%)
**RT**	I	176 (98)	2 (1)	1 (1)	179 (100)
	II	31 (94)	2 (6)	0	33 (100)
**CT**	I	7 (59)	4 (33)	1 (8)	12 (100)
	II	33 (66)	9 (18)	8 (16)	50 (100)
**CRT**	I	24 (96)	0	1 (4)	25 (100)
	II	28 (72)	7 (18)	4 (10)	39 (100)

Abbreviations: CR: Complete Remission. PR: Partial Remission. SD/PD: Stable Disease or Progressive Disease. Evaluation of response was missing for 3 patients. Total numbers and percentages are calculated horizontally (row directions).

### Relapse after response to initial treatment

Out of 330 patients who were initially treated and responded with at least stable disease (SD) or better, 165 patients (48%) relapsed. Relapse rates for stage I patients treated with RT, CT or CRT were 47%, 64% and 20%, respectively. Corresponding results for patients in stage II were 58%, 63% and 50%. Rate of relapse among patients with supradiaphragmatic disease was 55%, compared to 42% among patients with subdiaphragmatic disease (P = 0.02). Regardless of stage at time of initial diagnosis, disease involvement at time of first relapse was confined to one lymph node region for 56% of patients treated with RT alone, compared to only 34% for CT alone and 35% for CRT. Relapses were located partially or completely within initial radiation field in only 4 (2%) patients treated with RT or CRT. The location of relapse was close to or far from the primary radiation field for 29 (10%) and 98 (35%) of patients, respectively. Only 14 patients (5%) had isolated relapses close to the original radiation field.

In the OBS group 29 (46%) patients progressed (13 patients with primary stage I and 16 patients with stage II). Treatment was given to 26/29 patients at time of progression (CT = 15/26, CRT = 7/26 and RT = 4/26 patients). Progression involved more than 2 lymph node/extranodal regions in 7 out of 13 patients who progressed from stage I and in 12 out of 16 patients from stage II. One of the three patients in OBS group who progressed but never received treatment died of lymphoma 8 years after primary diagnosis. The other two patients were still alive at end of the study, 11 and 13 years after diagnosis, respectively.

### Overall Survival (OS)

**Fig 1:**
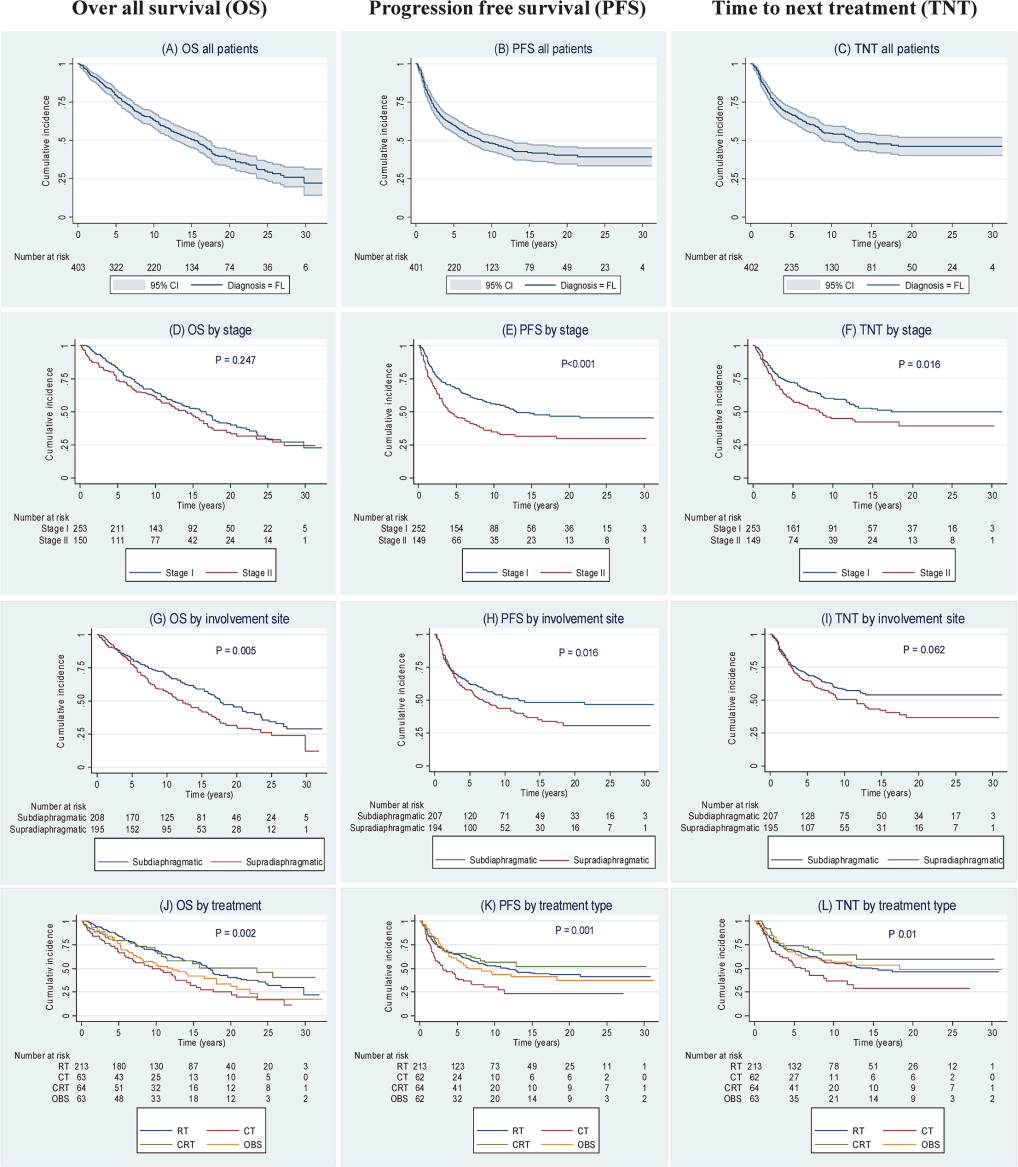
Kaplan-Meier survival curves showing overall survival (OS), progression free survival (PFS) and time to next treatment (TNT). Survival curves are illustrated as follows: (A) OS for all follicular lymphoma (FL) patients; (B) PFS for all FL patients; (C) TNT for all FL patients; (D) OS by stage; (E) PFS by stage; (F)TNT by stage; (G) OS by involvement site; (H) PFS by involvement site; (I) TNT by involvement site; (J) OS by treatment type; (K) PFS by treatment type; and (L) TNT by treatment type. Note: Pointwise confidence bands are not shown when survival curves overlap or run close to each other. P-values in (D) to (I) are from log-rank tests comparing the two groups, in (J) to (L) from generalized log-rank tests comparing all groups.

OS at 10, 15 and 20 years was 63% (95% CI: 58–68), 50% (95% CI: 45–55) and 38% (95% CI: 32–44), respectively (Fig 1A). There were no statistically significant differences in OS between stages I and II (P = 0.25) (Fig 1D). There was a significant difference between the treatment groups (P = 0.002) (Fig 1J). OS was significantly better in the cohort treated with RT compared to patients treated with CT (P < .01). There was a trend towards longer OS in the RT group compared to the OBS group (P = 0.054) but no difference between RT and CRT (P = 0.66). OS was significantly better for patients with subdiaphragmatic disease involvement (P<0.01) (Fig 1G), FLIPI score 0 (P<0.01), WHO performance status 0 (P<0.01), and age <61 years (P<0.01) compared to supradiaphragmatic disease, FLIPI score ≥1, WHO ≥1 and age ≥61 years. Within OBS group no significant difference were found between patients who underwent surgical tumor excision and those who did not (P = 0.74).

### Progression Free Survival (PFS)

PFS after 10-, 15- and 20 years were 48% (95% CI: 43–53), 42% (95% CI: 36–47), and 40% (95% CI: 34–45), respectively (Fig 1B). There was a significant difference in PFS between stage I and II in favor of stage I (P<0.01) (Fig 1E). There was a significant difference between the treatment groups (P = 0.001) (Fig 1K). Patients treated with RT had a more favorable PFS compared to patients treated with CT (P<0.01). PFS for the RT and CRT groups pooled together at 15 years was 46%. There were no significant differences in PFS between RT, CRT and OBS. Patients with subdiaphragmatic disease involvement had a significantly better PFS (P = 0.02) (Fig 1H). There was a trend for a better PFS for patients younger than 61 years at the time of diagnosis (P = 0.06).

The PFS for the OBS group show few early relapses, but later events result in an estimated PFS slightly below that of the RT and CRT groups. Within the OBS group patients who underwent complete surgical tumor excision had a superior PFS compared to those with asymptomatic residual disease (P = 0.03).

### Time to Next Treatment (TNT)

After 10, 15 and 20 years of follow-up 54% (95% CI: 49–60), 48% (95% CI: 42–54) and 46% (95% CI: 40–52) of patients had not received new treatment, respectively (Fig 1C).

Compared to stage II TNT was significantly longer for patients with stage I (P = 0.02) (Fig 1F). There was a significant difference between the treatment groups (P = 0.01) (Fig 1L). As for PFS, TNT was significantly shorter for patients treated with CT, compared to either RT (P = 0.01) or CRT (P<0.01) while TNT was similar in the RT, CRT and OBS groups. The proportion of patients who still not received new treatment 10 years following RT, CT, CRT and OBS were 56% (95% CI: 49–63), 36% (95% CI: 22–50), 64% (95% CI: 51–77) and 56% (95% CI: 43–69) respectively. Disease involvement in relation to diaphragm didn't have a significant impact on TNT (Fig 1I). No differences in TNT were found when comparing age groups (</≥61 years), sex, WHO status and FLIPI scores (data not shown). For patients in OBS group, surgical tumor excision did not have a significant statistical impact on TNT (P = 0.29).

### Primary extranodal lymphomas

Patients with primary extranodal FL (44 patients with stage I/extranodal and 19 patients with stage II/extranodal) had an OS, PFS and TNT survival curves which were not significantly different from the nodal lymphomas (P = 0.09, P = 0.65 and P = 0.70, respectively). This was also the case for the 18 patients with primary cutaneous FL.

### Multivariate analyses

We fitted separate Cox models for OS, PFS and TNT with age, gender, disease involvement in relation to diaphragm, WHO performance status, FLIPI, bulky (</≥ 6 cm in diameter), stage and type of initial treatment as covariates (Table 4). Overall mortality risk was significantly higher for those older than 61 years (Hazard ratio [HR] = 4.23; 95% CI: 2.36–7.57; P<0.01). Moreover, WHO performance status of 1 or higher and supradiaphragmatic disease depicted a higher mortality risk (HR = 1.38; 95% CI: 1.03–1.87; P = 0.03) and (HR = 1.39; 95% CI: 1.05–1.84; P = 0.02), respectively.

**Table 4:** Multivariate analyses.

			OS			PFS			TNT		
		No.	HR	95% CI for HR	P- value	HR	95% CI for HR	P- value	HR	95% CI for HR	P- value
**Gender**	Female	181	1			1			1		
	Male	211	1.29	0.98–1.69	0.07	1.23	0.98–1.63	0.15	1.06	0.78–1.44	0.71
**Age**	18–60	219	1			1			1		
	≥ 61	173	3.86	2.30–6.47	<0.01	1.61	0.98–2.65	0.06	1.31	0.77–2.23	0.32
**WHO**	0	250	1			1			1		
	≥ 1	142	1.38	1.03–1.87	0.03	1.13	0.82–1.56	0.43	1.15	0.81–1.65	0.42
**FLIPI**	0	171	1			1			1		
	1	188	1.05	0.62–1.76	0.87	0.80	0.50–1.31	0.37	0.85	0.51–1.42	0.54
	≥ 2	33	1.18	0.61–2.29	0.61	1.05	0.54–2.07	0.88	0.87	0.40–1.90	0.73
**Disease involvement**	Subdiaphragmatic	201	1			1			1		
	Supradiaphragmatic	191	1.39	1.05–1.84	0.02	1.44	1.07–1.93	0.01	1.42	1.02–1.96	0.03
**Stage**	I	246	1			1			1		
	II	146	1.12	0.80–1.55	0.51	1.70	1.22–2.36	<0.01	1.41	0.98–2.04	0.07
**Bulky**	Not bulky	341	1			1			1		
	Bulky (max diam ≥ 6cm)^a^	51	1.36	0.90–2.06	0.14	1.42	0.93–2.15	0.10	1.44	0.90–2.29	0.13
**Type of treatment**	RT	208	1			1			1		
	CT	61	1.49	0.98–2.27	0.06	1.28	0.83–1.98	0.27	1.34	0.83–2.17	0.24
	CRT	62	1	0.63–1.57	0.99	0.70	0.44–1.11	0.13	0.65	0.38–1.10	0.11
	OBS	61	1.05	0.72–1.52	0.80	0.99	0.66–1.48	0.95	0.89	0.56–1.41	0.63

Comments: 12 patients were excluded from multivariate analyses as they lack FLIPI score.

^a^ Maximum tumor diameter.

Increased risk for progression or death of lymphoma was found to be associated with stage II (HR = 1.70; 95% CI: 1.22–2.36; P<0.01) and supradiaphragmatic disease (HR = 1.44; 95% CI: 1.07–1.93; P = 0.01). The only significant risk factor for receiving next treatment was supradiaphragmatic disease (HR = 1.42; 95% CI: 1.02–1.96; P = 0.03).

### Second cancer

During follow-up, 72 patients (18%) were diagnosed with a second cancer and 4 and 2 patients with, third and fourth cancer, respectively. Types of second cancer are illustrated in S1 Table. The incidence of second cancer showed no statistically significant correlation to treatment given, stage of FL, gender, age or primary involvement site whether supra- or sub diaphragmatic (data not shown).

### Cause of death for patients and controls

By Dec 31 2012, 172 patients were still alive (43%). Cause of death for all patients and for subgroups receiving different types of primary treatment are shown in Table 5. Lymphoma was the most frequent cause of death within the group treated with CT (71% of deaths) compared to RT (38%), CRT (57%) and OBS (50%) (P<0.001). For patients with stage I, lymphoma was the cause of death in 54 cases (37%), compared to 59 cases (67%) for patients with stage II (P<0.001).

**Table 5:** Cause of death according to the type of treatment.

Treatment	Lymphoma	Other cancers	CVD	Other causes^a^	Total
	No. (%)	No. (%)	No. (%)	No. (%)	No. (%)
RT	43 (38%)	31 (27%)	20 (17%)	21 (18%)	115 (100%)
CT	33 (71%)	5 (11%)	4 (9%)	4 (9%)	46 (100%)
CRT	16 (57%)	6 (22%)	2 (7%)	4 (14%)	28 (100%)
OBS	21 (51%)	7 (17%)	7 (17%)	6 (15%)	41 (100%)

Comments:

^a^ Cause of death was unknown for 2 patients. Abbreviation: CVD: Cardiovascular disease. Total numbers and percentages are calculated horizontally (row directions).

**Fig 2:**
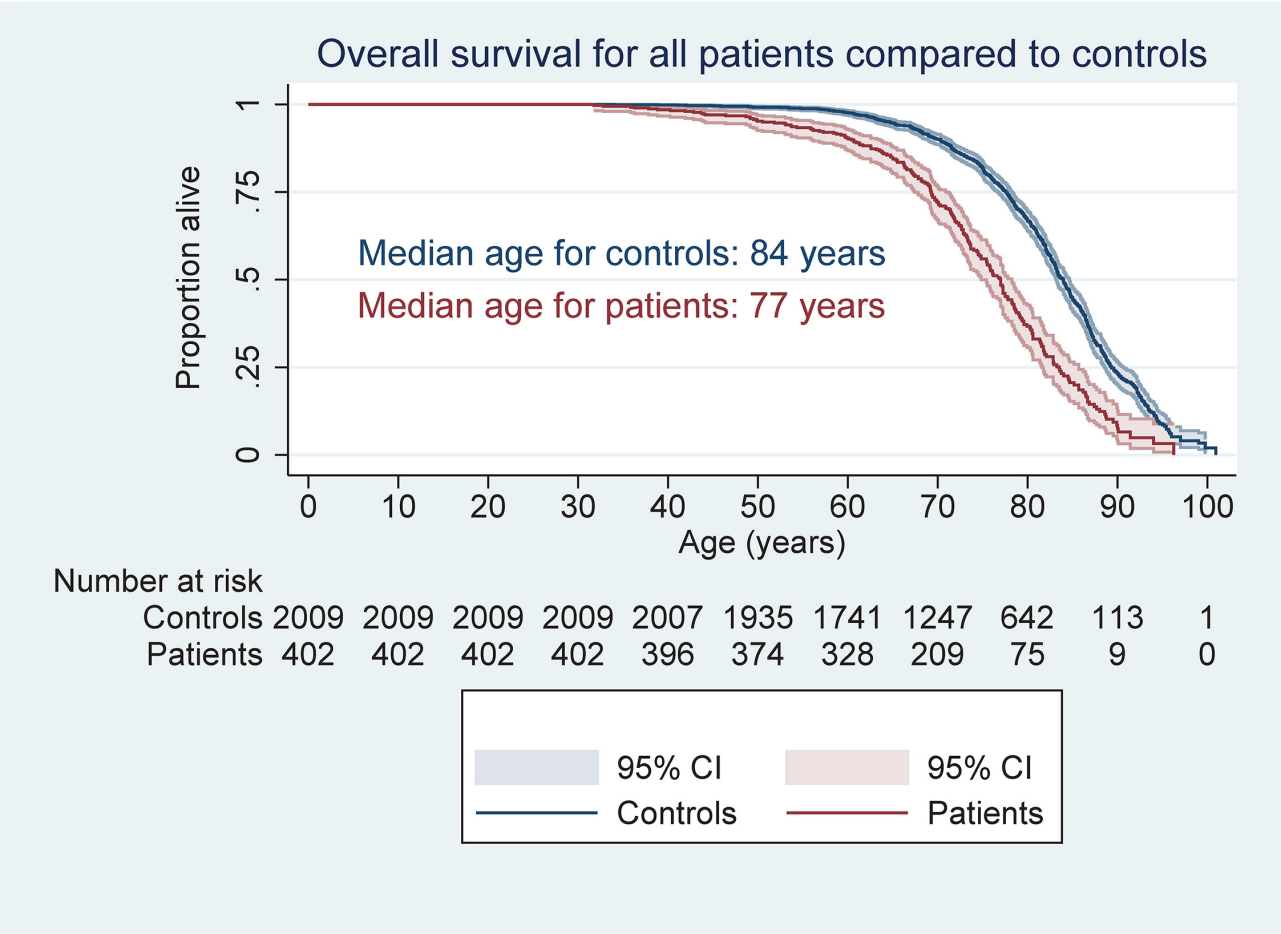
Overall survival for patients compared to controls.

OS for our patients was 7 years shorter compared with the age, sex and residency matched normal population. This difference was nearly equal for all age groups after the age of 60 years (Fig 2).

When the three main non-lymphoma causes of death were analyzed separately using the Kaplan-Meier survival curves, the cumulative incidence of non-lymphoma cancer related mortality was higher both for the whole study population and for RT treated patients compared to their matched controls. Cox regression with sex as additional covariate confirmed this results, with hazard ratio for death due to non-lymphoma cancer of 1.55 (95% CI: 1.02–2 .35; P = 0.04) for RT patients and 1.66 (95% CI: 1.14–2.42; P<0.01) RT+CRT patients compared to controls. No significant increased risk of non-lymphoma cancer was observed for the other treatment groups. The risk of death from CVD or other causes than cancer was not elevated in any of the treatment groups, compared to controls.

## Discussion

Outcome for early stage FL has been reported previously [2–8, 19], all in studies with shorter follow-up than ours. Additionally, the large number of patients and the population based recruitment of patients distinguish our study from the others. Thus, the disadvantage of selection and information bias which is often a problem in retrospective studies was minimized. Since every case of malignant disease in Norway is entered into Cancer Registry Norway, we were able to obtain detailed information about secondary cancers. Moreover, information about date of birth, sex and residency for every individual in Norway is registered in Statistics Norway, and we could therefore obtain five sex- and age matched controls from the normal population for each patient in our study.

Estimated ten-year OS reported in previous studies has ranged between 42% and 78% [2–8] compared to 63% in our study. We did not observe any survival benefit for patients treated with CRT compared to patients who received RT alone or no therapy. This is in accordance with results from other groups [7, 9, 20]. Although PFS and TNT were prolonged in the group treated with RT compared with CT in univariate analysis, the survival advantage was not sustained when adjusting for age, gender, WHO status, FLIPI score, bulky disease, involvement in relation to diaphragm and stage.

Other studies have suggested that a watch and wait strategy does not compromise survival for selected patients with early stage FL [21, 22]. In our study patients who were observed without therapy had similar OS and TNT compared to other treatment groups. PFS for patients in whom the tumor was completely removed by surgical excision was more favorable compared to patients with residual disease. However, we did not observe any difference between these two groups in regard to OS or TNT. The favorable outcome for the OBS cohort suggests that observation without treatment is an acceptable initial strategy for selected patients; however, the possibly elevated number of late relapses underlines the necessity of close long-term follow-up.

In accordance with the literature [3, 23, 24], our study revealed a high rate of CR (97%) after RT. Adding CT neither improved response rates nor decreased relapse rates. Between four and five out of ten patients treated with RT were relapse free by the end of the study. As only very few relapses occurred inside the radiation field, and isolated relapses close to the radiation field also were uncommon, the radiation dose and volume administered seem at least adequate. An increased risk of death from other cancers, however, is a matter of concern. We have reduced the radiation dose further after the study period to 2 Gy x 12 based on a randomized trial [25].

The majority of our patients were treated before rituximab (R) became an option for patients with FL. Martinelli et al. reported results from a clinical first line randomized trial for 202 patients, most with advanced stage FL disease, showing that one of three patients in the R maintenance arm were event free after 9–10 years, but with no indication of plateau in the survival curve. [26]. A more recent retrospective study in patients with stage I FL did not reveal a survival difference in terms of OS when comparing R-CT, R alone, R-CRT, RT alone or no treatment [20]. However, in patients including PET-scan for staging, they did report superior PFS for the R-CRT group over the RT alone group. As follow-up time for this study was relatively short, further data is needed to address the issue of combined modality treatment and the role of rituximab in early stage FL.

Since our study included many patients diagnosed and treated several decades ago, there are some limitations and confounding factors. We did not apply PET for staging in this study, as recently recommended [27]. Central histopathological review was not mandatory in the study. However, all specimens were evaluated in our hospital which has been a reference center for lymphoma pathology in Norway since the 1970`s. Causes of death for about half of the patients who died were collected from Statistics Norway and based on death reports from local hospitals or general practitioners. For individuals with a history of lymphoma, lymphoma as a cause of death might have been overestimated. Another limitation is that the staging investigations varied over time with some of the early patients were staged with a chest x-ray and computed tomography only of abdomen and pelvis. Thus, 28% of the patients diagnosed before 1986 did not have computed tomography of the chest, and a fraction of these patients with disease below the diaphragm from that period (15% of all patients) may have had a stage III disease. It has been shown that a more rigorous staging of early stage FL affects the outcome [20]. Further, computed tomography was not performed regularly during the long follow up period, which may have postponed the date of relapse / progression in the PFS analyses for some patients.

We have no control group from the general population with figures on incidence of second cancer and CVD. As disease incidence may be related to and precede mortality, such data would be of interest, but were beyond the scope of this project when planned.

Different approaches to the multivariate analyses were considered. As indicated above, the OBS group had a time pattern of relapses differing somewhat from the other groups, thus challenging the proportional hazard assumption. However, results from the Cox regression were similar when including or excluding the OBS group. Other studies have previously shown that increasing age at the time of diagnosis has an adverse prognostic impact on OS [2–4, 6]. This was confirmed both by univariate and multivariate analyses in our study. Additionally, WHO performance status ≥1 and supradiaphragmatic disease were associated with shorter OS. Contrary to other reports [3, 6], we could not find that stage had any impact on OS in multivariate analysis. In one of these studies [6] 25% of patients underwent staging laparotomy and two thirds of patients were treated with either extended field RT or total lymphoid irradiation which is associated with long term morbidity and mortality.

Supradiaphragmatic disease involvement was also a significant risk factor for PFS and TNT in both uni- and multivariate analyses. These differences could not be explained by incidences of secondary cancers or death from CVD. The majority of deaths for patients with supradiaphragmatic disease (52%) were due to lymphoma compared to 44% for patients with subdiaphragmatic disease. This could be due to a more disseminated disease above the diaphragm than recognized by staging.

In accordance to previous reports [4, 28] lymphoma was the main cause of death for all treatment groups. The hazard ratio of second cancer deaths was significantly elevated for patients treated with RT, with or without CT compared to non-lymphoma deaths in the general population. Our current standard RT dose of 24 Gy compared to 30–40 Gy for the study patients might reduce the risk of death from second cancers. When comparing patients and controls, we also tested competing risk models using the Fine and Gray approach [29, 30]. This approach confirmed a higher occurrence of deaths due to secondary cancers compared to other non-lymphoma causes of death. Since this comparison primarily aims at revealing etiology, we followed the advice of Andersen et al for such studies and reported the results of the Cox regressions [31].

In conclusion, we report outcome for patients with limited stage follicular lymphoma patients with long follow-up for a population-based cohort and with differentiated treatment approach based on clinical features. PFS at the median follow-up time of 15 years for all patients who received RT was 46%. However, an increased mortality from secondary cancers supports the current practice of a lower radiotherapy dose. A group of selected patients without cancer related symptoms may safely be recommended initial observation. Long term data about the role of rituximab in treatment of early stage FL are yet not available and this should be an issue for future studies.

## Supporting Information

S1 Table Types and frequencies of second, third and fourth cancer in all FL patients. (DOCX)
