# Supplementary material for: Radiotherapy Compared to Other Strategies in the Treatment of Stage I/II Follicular Lymphoma: A Study of 404 Patients with a Median Follow-Up of 15 Years
Source: PLoS One. 2015 Jul 6;10(7):e0131158. doi: 10.1371/journal.pone.0131158 (PMC4492987; doi:10.1371/journal.pone.0131158)
Supplement: S1 Table — (DOCX) [file pone.0131158.s001.docx]

**S1 Table: Types and frequencies of second, third and fourth cancer in all FL patients.**

| Second Cancer | | Third Cancer | | Fourth Cancer | |
| --- | --- | --- | --- | --- | --- |
| Type of cancer | No. | Type of cancer | No. | Type of cancer | No. |
| Colorectal cancer | 11 | Squamous cell carcinoma of skin | 2 | Hodgkin's lymphoma | 1 |
| Prostate cancer | 11 | AML | 1 | Kaposi sarcoma | 1 |
| Lung cancer | 8 | Colorectal cancer | 1 |  |  |
| Breast cancer | 5 | Multiple myeloma | 1 |  |  |
| Squamous cell carcinoma of skin | 4 | Prostate cancer | 1 |  |  |
| Pancreas cancer | 3 |  |  |  |  |
| Uterine cancer | 3 |  |  |  |  |
| Urothelial carcinoma | 3 |  |  |  |  |
| Cancer origo incerta | 2 |  |  |  |  |
| Chondrosarcoma | 2 |  |  |  |  |
| Cerebral Glioblastoma | 2 |  |  |  |  |
| Malignant melanoma of skin | 2 |  |  |  |  |
| Thyroid cancer | 2 |  |  |  |  |
| Gastric cancer | 2 |  |  |  |  |
| Acute lymphocytic leukemia | 1 |  |  |  |  |
| Acute myeloid leukemia | 1 |  |  |  |  |
| Adrenal cancer | 1 |  |  |  |  |
| Cholangiocarcinoma | 1 |  |  |  |  |
| Chronic lymphocytic leukemia | 1 |  |  |  |  |
| Chronic myeloid leukemia | 1 |  |  |  |  |
| Hodgkin's lymphoma | 1 |  |  |  |  |
| Kaposi Sarcoma | 1 |  |  |  |  |
| Multiple myeloma | 1 |  |  |  |  |
| Ovarian cancer | 1 |  |  |  |  |
| Peritoneal cancer | 1 |  |  |  |  |
| Squamous cell carcinoma of gingiva | 1 |  |  |  |  |
| Total | 72 | Total | 6 | Total | 2 |
